# Comparing Dydrogesterone Versus Medroxyprogesterone in Progestin-Primed Ovarian Stimulation (PPOS) for Patients Undergoing In Vitro Fertilization/Intracytoplasmic Sperm Injection: A Systematic Review

**DOI:** 10.7759/cureus.85959

**Published:** 2025-06-13

**Authors:** R Muharam, Alisha Nurdya Nurdya, Edward C Yo, Kanadi Sumapraja, Achmad Kemal Harzif, Mila Maidarti, Budi Wiweko, Andon Hestiantoro

**Affiliations:** 1 Reproductive Immunoendocrinology Division, Department of Obstetrics and Gynecology, Faculty of Medicine, University of Indonesia, Jakarta, IDN; 2 Reproductive Endocrinology and Infertility, Dr. Cipto Mangunkusumo National Central General Hospital, Jakarta, IDN

**Keywords:** dydrogesterone, intracytoplasmic sperm injection, in vitro fertilization, medroxyprogesterone, ovarian hyperstimulation, progestin primed ovarian stimulation

## Abstract

Dydrogesterone (DYG) in the progestin-primed ovarian stimulation (PPOS) protocol is an alternative progestin with weaker pituitary suppression than medroxyprogesterone acetate (MPA) in women with normal ovulation. However, the endocrinological characteristics, oocyte retrieval, and pregnancy outcomes of DYG in PPOS patients undergoing in vitro fertilization (IVF) remain unclear. This systematic review aimed to compare the efficacy of DYG and MPA in PPOS protocols in IVF/intracytoplasmic sperm injection (ICSI) cycles. Studies published between 2018 and 2024 were identified through PubMed and the Cochrane Library. After screening and applying the eligibility criteria, only full-text articles directly comparing DYG and MPA in PPOS protocols for IVF/ICSI were included. A total of three studies involving 1,172 patients were analyzed. Both DYG and MPA effectively suppressed premature luteinizing hormone (LH) surges, with no significant differences in the oocyte yield, fertilization rates, or clinical pregnancy rates. DYG was associated with slightly higher post-trigger follicle-stimulating hormone (FSH) levels and lower LH levels compared to MPA, while both groups showed similar estradiol and progesterone trends. Some studies reported significantly lower gonadotropin requirements in the DYG group. Pregnancy outcomes, including biochemical and clinical pregnancy rates, implantation, and miscarriage, were comparable between groups. These findings indicate that both DYG and MPA are effective and safe for LH surge suppression in PPOS protocols, with DYG potentially offering a more physiologic hormonal profile and reduced gonadotropin use. Further randomized controlled trials are recommended to validate these results.

## Introduction and background

Infertility affects an estimated 10% of couples of childbearing age worldwide, making it a significant global health concern. Assisted reproductive technologies (ART), especially intracytoplasmic sperm injection (ICSI) and in vitro fertilization (IVF), have become the most effective interventions for overcoming various causes of infertility, including tubal, anovulatory, and unexplained etiologies [1,2]. The cornerstone of a successful IVF/ICSI cycle is controlled ovarian stimulation (COS), which aims to recruit multiple follicles in a single cycle to maximize the number of retrievable oocytes. However, COS is not without challenges; most notably, the risk of a premature luteinizing hormone (LH) surge, which can lead to premature ovulation, cycle cancellation, and reduced IVF success rates [3]. 

Various strategies have been developed to prevent premature LH fluctuations during COS. Traditionally, gonadotropin-releasing hormone (GnRH) analogues, either antagonists or agonists, have been employed for this purpose. However, in recent years, the progestin-primed ovarian stimulation (PPOS) protocol has emerged as a promising alternative. Introduced in 2015, PPOS involves the administration of oral progestins, such as medroxyprogesterone acetate (MPA) or dydrogesterone (DYG), in combination with human menopausal gonadotropin (hMG). This approach effectively inhibits the LH surge while offering the convenience of oral medication and potentially reducing costs compared to the GnRH analogues [4].

The PPOS protocol offers several clinical and practical advantages. By replacing injectable GnRH analogues with oral progestins, PPOS simplifies treatment regimens and enhances patient compliance. Moreover, progestins act directly at the hypothalamic-pituitary axis to inhibit LH secretion, thereby creating a more stable and favorable hormonal environment for follicular development. Suppression of premature LH surges also ensures optimal timing for ovulation triggering, increasing the number and maturity of retrieved oocytes and minimizing cycle cancellations.

Among progestins, MPA has been extensively studied and widely used in PPOS protocols. Its efficacy in preventing LH surges and supporting follicular growth has been well documented [5]. However, MPA has been associated with stronger pituitary suppression, which may necessitate higher gonadotropin doses and longer stimulation durations, potentially impacting both cost-effectiveness and patient burden [6].

In contrast, DYG, a synthetic progestin structurally similar to natural progesterone, has emerged as a potential alternative with a milder suppressive effect on the pituitary [7]. This property may reduce the total dosage of gonadotropins required and lead to a more physiologic ovarian response. Additionally, DYG’s lack of androgenic, glucocorticoid, and estrogenic activity makes it a favorable candidate for use in ART. Preliminary studies have suggested that DYG may offer comparable efficacy to MPA in terms of oocyte retrieval and pregnancy outcomes, while potentially improving tolerability and ovarian responsiveness [8,9].

Despite these developments, the availability of evidence comparing DYG and MPA in PPOS protocols remains limited and inconsistent. Most existing studies have focused on specific subpopulations, such as women with polycystic ovary syndrome (PCOS) or normal ovarian reserve, while less is known about their comparative effectiveness in the general IVF/ICSI population.

This systematic review aims to comprehensively assess the comparative efficacy of DYG versus MPA in PPOS protocols for patients undergoing IVF/ICSI, across varying levels of ovarian reserve. This work was previously presented as an oral presentation at the 2024 Asia Pacific Initiative on Reproduction (ASPIRE) on May 25, 2024.

## Review

Materials and methods

Study Design and Population

This study was designed as a systematic review following the Preferred Reporting Items for Systematic Reviews and Meta-Analyses (PRISMA) guidelines [10]. The review aimed to compare the efficacy and safety of DYG versus MPA in PPOS protocols among women undergoing IVF/ICSI.

*PICO Framework *[11]

To guide the review, the following PICO components were defined:

Population (P): Women undergoing IVF or ICSI treatment

Intervention (I): Use of DYG in a PPOS protocol

Comparison (C): Use of MPA in a PPOS protocol

Outcomes (O): Clinical outcomes such as the number of oocytes retrieved, embryo quality, pregnancy rates, and live birth rates

Search Strategy

A comprehensive search was conducted across two major biomedical databases, PubMed and Cochrane Library, to identify relevant studies published between January 2018 and April 2024. The search strategy utilized a combination of Medical Subject Headings (MeSH) terms and free-text keywords, including but not limited to "Dydrogesterone", "Medroxyprogesterone Acetate", "Progestin-Primed Ovarian Stimulation", "PPOS", "IVF", "ICSI", "controlled ovarian stimulation". Boolean operators (AND/OR) were used to combine terms appropriately. Reference lists of included studies were also screened manually to identify any additional eligible studies not captured through database searches.

Inclusion and Exclusion Criteria

The inclusion criteria were original studies published in English, involving human participants undergoing IVF/ICSI treatment, and with a direct comparison of DYG and MPA within a PPOS protocol.

Studies were excluded if they were case reports, editorials, or narrative reviews, lacking a direct comparison between DYG and MPA, and were focused on non-PPOS stimulation protocols.

Study Selection and Variability

After removing duplicates, titles and abstracts were independently screened for relevance by the second and third authors. Full-text articles were then reviewed to assess eligibility. Any disagreements were resolved through discussion among all the three authors.

The included studies varied in study design, comprising both randomized controlled trials (RCTs) and retrospective cohort studies. These methodological differences were considered when assessing the quality and interpretability of the data. Subgroup analyses and sensitivity assessments were performed where applicable to account for design-related heterogeneity.

Outcome Measures and Definitions

The primary outcome measure in this systematic review was the number of oocytes retrieved, consistently reported across all included studies. This parameter served as a key indicator of ovarian response and reflected the effectiveness of the PPOS protocol when using either DYG or MPA.

Secondary outcome measures included a range of variables assessing treatment efficacy and reproductive potential, such as oocyte retrieval rate (%), fertilization rate (%), viable embryo rate per oocyte retrieved (%), and cycle cancellation rate (n, %). In addition, hormonal profiles, specifically levels of FSH, LH, estradiol (E2), and progesterone (P), were evaluated to assess the degree of LH suppression and overall endocrine response throughout the controlled ovarian stimulation.

All outcome measures were defined and interpreted according to standard clinical practice and as reported within the methodologies of the respective included studies.

Risk of Bias Assessments

Each included study was independently evaluated using the Cochrane Risk of Bias Assessment Tool [12] by the second and third author. The tool covers seven key domains: (1) Random sequence generation (2) Allocation concealment (3) Blinding of participants and personnel (4) Blinding of outcome assessment (5) Incomplete outcome data (6) Selective reporting and (7) Other sources of bias. Each domain was categorized as having low risk, unclear risk, or high risk depending on the reporting quality and methodological rigor. 

Statistical Analysis

Due to the heterogeneity of included studies and variations in outcome reporting, data were synthesized qualitatively. Extracted data from full texts, tables, graphs, and references were compiled to allow for comparative analysis between DYG and MPA groups. Descriptive statistics were employed to compile the findings, and where applicable, outcome trends were presented in tabular or graphical form. Meta-analysis was not performed due to limited comparable datasets across studies.

Results

**Figure 1:**
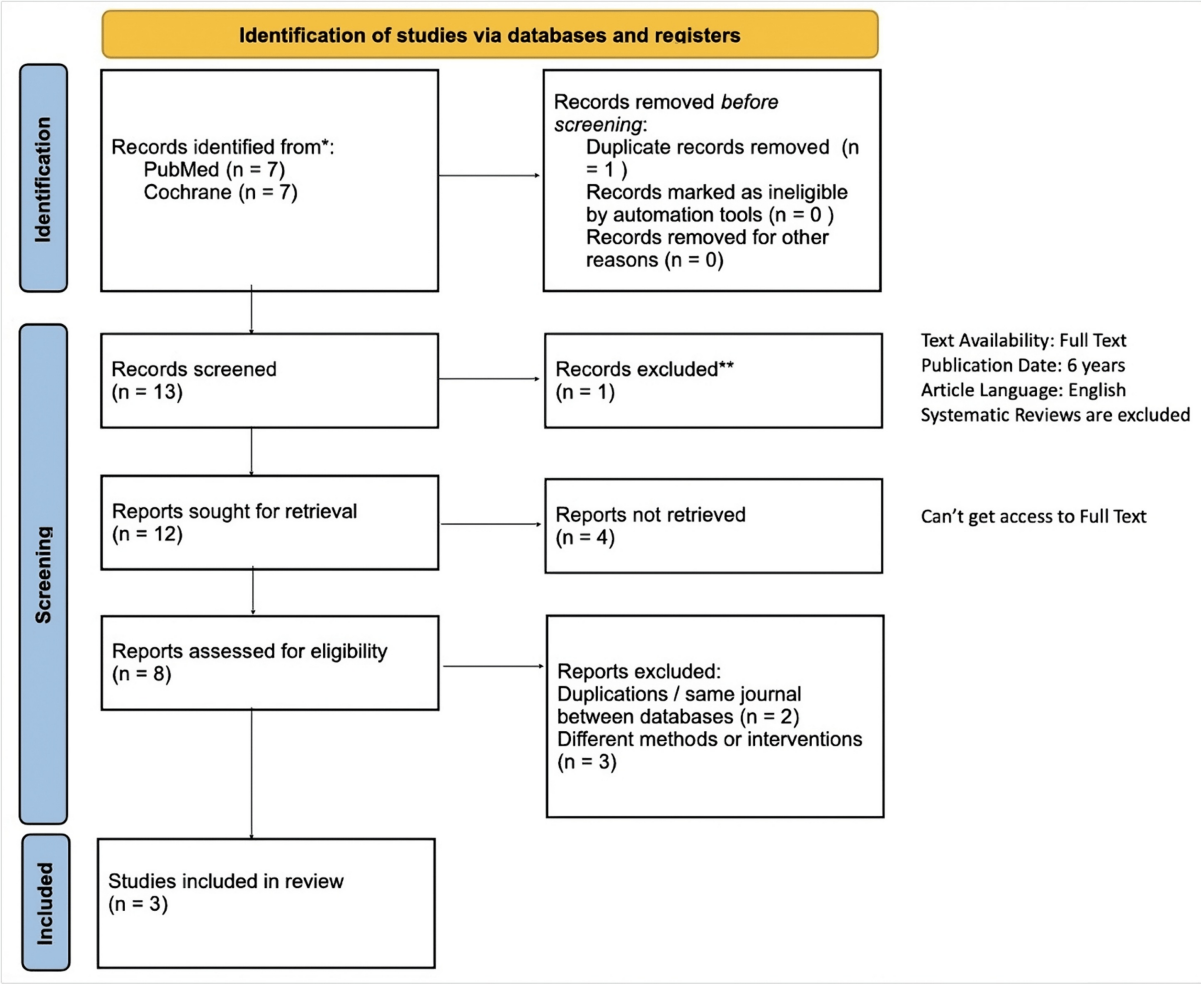
PRISMA flow diagram of searching on selected studies *Studies identified from specific databases; **Studies excluded after full-text review for not meeting the inclusion criteria.

This systematic review initially identified 14 records through comprehensive searches of the PubMed and Cochrane databases. After removal of duplicates (n=1), 13 unique studies remained for screening. During the screening phase, one systematic review (n=1) was excluded due to study design. After evaluating the full-text eligibility, only studies directly comparing DYG and MPA within the PPOS protocol for IVF/ICSI patients were included. Ultimately, three studies published between 2018 and 2024 met the inclusion criteria. The detailed process of research identification, screening, eligibility assessment, and final inclusion is shown in the PRISMA flow diagram (Figure 1).

These studies collectively evaluated 1,172 patients experiencing IVF/ICSI treatment using PPOS with either DYG or MPA in combination with hMG. An overview of the characteristics of the included studies is compiled in Table 1.

**Table 1:** Summary of included studies POR: Poor ovarian reserve; PCOS: Polycystic ovary syndrome; hMG: Human menopausal gonadotropin; DYG: Dydrogestrone; MPA: Medroxyprogesterone; LH: Luteinizing hormone

Study	Study design	Population (n)	Intervention (DYG/MPA)	Primary outcome	Main findings
Zhang et al., 2021 [13]	Retrospective cohort study	236 (POR)	hMG + DYG (n=118) vs hMG + MPA (n=118)	No. of oocytes retrieved	No significant differences in oocyte yield, embryo development, or pregnancy outcomes. Both groups achieved LH suppression.
Huang et al., 2019 [14]	Retrospective cohort study	420 (PCOS)	hMG + DYG (n=105) vs hMG + MPA (n=315)	No. of oocytes retrieved	Comparable clinical outcomes, LH suppression effective in both groups; DYG group required less hMG.
Yu et al., 2018 [15]	Randomized controlled trial	516 (Normal ovarian reserve)	hMG + DYG (n=260) vs hMG + MPA (n=256)	No. of oocytes retrieved	No significant differences in oocyte yield, fertilization, or pregnancy outcomes; DYG is a suitable alternative.

The primary outcome measure, the number of oocytes retrieved, was reported consistently across all three included studies. In Zhang et al. [13], the mean number of oocytes retrieved was slightly lower in the DYG group compared to the MPA group (3.9±2.8 vs. 4.5±3.0), though the difference did not reach statistical significance (P=0.10). Furthermore, Huang et al. [14] and Yu et al. [15] observed comparable oocyte yields between the two groups (16.1±6.5 vs. 15.1±10.0, P=0.342; and 10.8±6.3 vs. 11.1±5.8, P=0.33, respectively), indicating no substantial advantage of either progestin in terms of ovarian response.

Secondary outcomes also revealed no significant differences in the treatment efficacy between DYG and MPA. Oocyte retrieval rates were similar across studies: 74.4% vs. 71.0% (P=0.14) in Zhang et al. [13], 63.1% vs. 62.5% (P=0.508) in Huang et al. [14], and 74.3% vs. 75.0% (P=0.69) in Yu et al. [15]. The viable embryo rate per oocyte retrieved ranged from 34.3% to 50.5% in the DYG groups and from 35.6% to 46.3% in the MPA groups, with no statistically significant differences observed [14,15]. Similarly, fertilization rates did not differ significantly between DYG and MPA across all studies (e.g., 56.9% vs. 55.5%; P=0.26 in Zhang et al. [13]).

With respect to gonadotropin usage, Huang et al. [14] revealed a significantly lower total hMG dose in the DYG group (1710.7±431.6 vs. 1891.3±402.2 IU, P<0.001), suggesting a potential efficiency advantage for DYG in terms of medication burden. However, this trend was less pronounced in Yu et al. [15] (1912.2±242.1 vs. 1959.1±236.4 IU, P=0.04) and not significant in Zhang et al. [13] (P=0.91). The duration of stimulation remained consistent across groups in all studies, with no significant differences.

Cycle cancellation rates were also comparable between groups where reported. Zhang et al. [13] noted a slightly lower cancellation rate in the DYG group (16.9% vs. 22.0%, P=0.32), while Yu et al. [15] found a numerically lower, but not a statistically significant rate in the DYG group (6.9% vs. 10.2%, P=0.19). Cancellation rates were not reported in Huang et al. [14] (Table 2).

**Table 2:** Cycle characteristics of women treated with the hMG+DYG and hMG+MPA protocols hMG: Human menopausal gonadotropin; DYG: Dydrogestrone; MPA: Medroxyprogesterone

Study	Outcome measure	DYG group	MPA group	P-value
Zhang et al., 2021 [13]	Dosage of gonadotropins (IU)	2728.6±873.9	2715.7±926.3	0.91
	Duration of ovarian stimulation (days)	10.0±2.7	9.8±2.7	0.60
	No. of oocytes retrieved	3.9±2.8	4.5±3.0	0.10
	Oocyte retrieved rate (%)	74.4±16.8	71.0±20.0	0.14
	Viable embryo rate per oocyte retrieved (%)	50.5±24.0	46.3±24.2	0.16
	Fertilization rate (%)	56.9±21.5	55.5±23.7	0.26
	Cancellation rate, n (%)	20/118 (16.9)	26/118 (22.0)	0.32
Huang et al., 2019 [14]	hMG dose (IU)	1710.7±431.6	1891.3±402.2	<0.001
	hMG duration (days)	9.1±1.6	9.0±1.6	0.670
	No. of oocytes retrieved	16.1±6.5	15.1±10.0	0.342
	Oocyte retrieval rate (%)	63.1±15.4	62.5±22.6	0.508
	Viable embryo rate per oocyte retrieved (%)	34.3±19.2	36.1±22.4	0.556
	Fertilization rate (%)	76.2±19.7	75.3±21.1	0.760
	Cancellation rate, n (%)	-	-	-
Yu et al., 2018 [15]	Total hMG dose (IU), mean (SD)	1912.2 (242.1)	1959.1 (236.4)	0.04
	Days of stimulation, mean (SD)	8.5 (1.1)	8.7 (1.1)	0.06
	No. of oocytes retrieved	10.8 (6.3)	11.1 (5.8)	0.33
	Oocyte retrieval rate (%), mean (SD)	74.3 (19.6)	75.0 (19.5)	0.69
	Viable embryo rate per oocyte retrieved (%)	1052/2815 (37.4)	1009/2837 (35.6)	0.16
	Fertilization rate (%), mean (SD)	75.1 (22.0)	72.0 (24.4)	0.21
	Cancellation rate, n (%)	18/260 (6.9)	26/256 (10.2)	0.19

**Figure 2:**
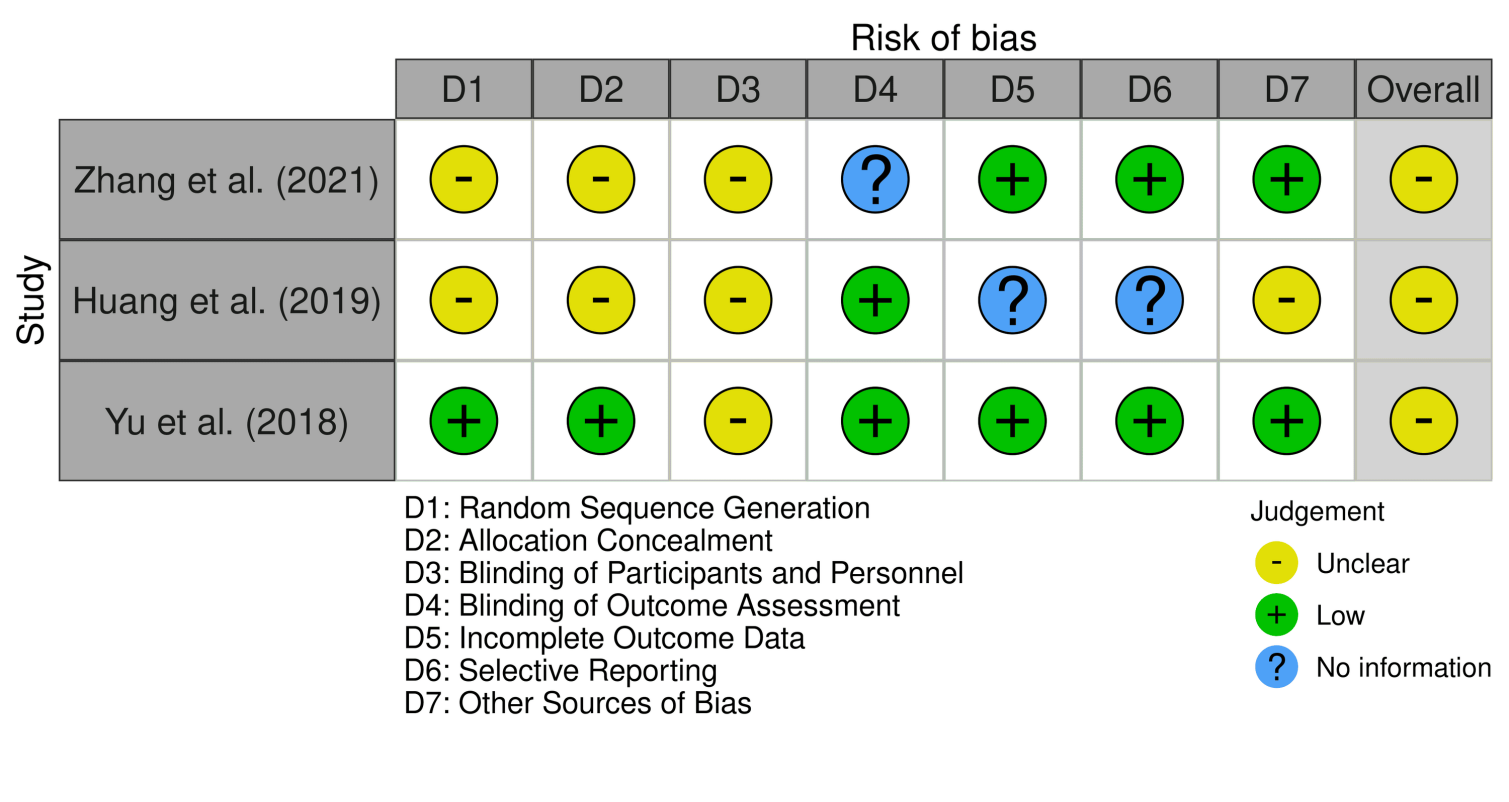
Risk of bias summary [13-15]

All the three included studies showed some concerns regarding risk of bias, mainly due to insufficient reporting on randomization methods and blinding procedures. Zhang et al. [13] and Huang et al. [14] did not fully describe the random sequence generation or allocation concealment, and were open-label with unclear blinding of outcome assessors. Yu et al. [15] adequately addressed randomization but lacked clarity on the blinding of the participants and outcome assessors. Risk of incomplete outcome data was generally low, except for Huang et al. [14], which lacked sufficient information. Selective reporting and other biases were mostly low risk, although Huang et al. [14] showed some concerns due to its retrospective design. A detailed evaluation of the risk of bias is presented in Figure 2.

Hormone Profile

The hormonal profiles of patients undergoing PPOS using DYG or MPA were evaluated across the included studies [13-15]. Overall, both progestins demonstrated effective suppression of premature LH surges, with no significant differences reported in surge incidence between groups in Zhang et al. [13] (2.5% vs. 1.7%, P=0.65). There were no reported cases of premature LH surge in the studies by Huang et al. [14] or Yu et al. [15]. LH levels were higher in the DYG group than in the MPA group on the trigger day in the study by Zhang et al. [13], even though the difference was not statistically significant (P=0.11). Nevertheless, post-trigger LH levels rose significantly in both groups, with the MPA group exhibiting markedly higher levels than the DYG group (60.3±30.8 vs. 38.1±21.4 IU/L, P<0.01). Huang et al. [14] similarly reported higher LH levels in the DYG group on the trigger day (2.74±1.50 IU/L, P<0.001), while Yu et al. [15] observed consistent LH suppression before the trigger and significant elevation post-trigger in both groups (P<0.001).

FSH profiles showed comparable patterns across studies, with levels rising after hMG initiation, plateauing during mid-stimulation, and increasing sharply post-trigger. Notably, Yu et al. [15] reported higher post-trigger FSH levels in the DYG group compared to the MPA group (26.5±6.3 vs. 24.7±6.4 mIU/mL, P<0.01), while Huang et al. [14] discovered no significant difference between the groups. Zhang et al. [13] did not report FSH values.

E2 levels increased gradually in both groups throughout ovarian stimulation in all three studies, with no significant differences between DYG and MPA protocols. P levels remained low during stimulation and rose significantly after triggering in both groups, again with no notable between-group differences (Table 3).

**Table 3:** The pregnancy outcomes for the first frozen embryo transfer cycles in the two groups DYG: Dydrogestrone; MPA: Medroxyprogesterone

Study	Pregnancy outcomes	DYG group	MPA group	P-value
Zhang et al., 2021 [13]	No. of patients	-	-	
	No. of cycles	66	87	
	Pregnancy outcomes per cycle, n/N (%)			
	Biochemical pregnancy rate	28/66 (42.4)	30/87 (34.5)	0.32
	Clinical pregnancy rate	24/66 (36.4)	27/87 (31.0)	0.49
	Implantation rate			
	Miscarriage rate	3/24 (12.5)	8/27 (29.6)	0.14
	Multiple pregnancy rate	-	-	
	Ectopic pregnancy rate	-	-	
Huang et al., 2019 [14]	No. of patients	95	261	
	No. of cycles	154	367	
	Pregnancy outcomes per cycle, n/N (%)			
	Biochemical pregnancy rate	90/154 (58.4)	238/367 (64.9)	0.167
	Clinical pregnancy rate	81/154 (52.6)	219/367 (59.7)	0.136
	Implantation rate	107/275 (38.9)	305/677 (45.1)	0.083
	Miscarriage rate	12/81 (14.8)	37/219 (16.9)	0.665
	Multiple pregnancy rate	22/81 (27.2)	81/219 (37.0)	0.112
	Ectopic pregnancy rate	2/81 (2.5)	7/219 (3.2)	1.000
Yu et al., 2018 [15]	No. of patients	217	212	
	No. of cycles	-	-	
	Pregnancy outcomes per cycle, n/N (%)			
	Biochemical pregnancy rate	136/217 (62.7)	138/212 (65.1)	0.60
	Clinical pregnancy rate	125/217 (57.6)	132/212 (62.3)	0.33
	Implantation rate	163/407 (40.0)	183/399 (45.9)	0.10
	Miscarriage rate	8/125 (6.4)	13/132 (9.8)	0.32
	Multiple pregnancy rate	38/125 (30.4)	53/132 (40.2)	0.10
	Ectopic pregnancy rate	6/125 (4.8)	6/132 (4.5)	0.92

Pregnancy and Neonatal Outcomes

Pregnancy outcomes following the first frozen embryo transfer (FET) cycle were assessed across the three included studies [13-15]. As shown in Table 3, Zhang et al. [13] reported no statistically significant differences in biochemical pregnancy rates (42.4% vs. 34.5%, P=0.32) or clinical pregnancy rates (36.4% vs. 31.0%, P=0.49) between the DYG and MPA groups.

A lower miscarriage rate was observed in the MPA group (29.6%) compared to the DYG group (12.5%), although this did not reach statistical significance (P=0.14). Similarly, Huang et al. [14] found comparable outcomes between groups, including implantation rate (38.9% vs. 45.1%, P=0.083), clinical pregnancy rate (52.6% vs. 59.7%, P=0.136), and miscarriage rate (14.8% vs. 16.9%, P=0.665). Yu et al. [15] also reported similar biochemical (62.7% vs. 65.1%, P=0.60) and clinical pregnancy rates (57.6% vs. 62.3%, P=0.33), with no significant differences in miscarriage or ectopic pregnancy rates.

Discussion

Across all studies, both DYG and MPA effectively suppressed premature LH surges, with no significant difference in incidence. This confirms the clinical viability of DYG as an alternative to MPA in PPOS protocols. While MPA is well established, DYG may offer certain advantages due to its milder suppression of the hypothalamic-pituitary axis, potentially leading to a more physiologic endocrine environment.

Ovarian Response and Embryological Outcomes

The primary outcome, number of oocytes retrieved, was consistently comparable between DYG and MPA groups. Similarly, secondary outcomes such as oocyte retrieval rate, fertilization rate, viable embryo rate per oocyte, and cycle cancellation rate showed no statistically significant differences. These findings indicate that both DYG and MPA are equally effective in supporting follicular development and embryo generation, regardless of the underlying ovarian reserve.

Interestingly, two studies (Huang et al. and Yu et al. [14,15]) showed lower total hMG consumption in the DYG group, with one study demonstrating a statistically significant reduction. This suggests that DYG may facilitate a more efficient ovarian stimulation, potentially reducing medication burden and treatment costs without compromising clinical outcomes. These findings align with results reported by Lokshin et al. [9], which also observed lower gonadotropin consumption in DYG-based protocols compared to MPA. This consistency across studies underscores a shared interest in dose efficiency and cost-effectiveness [9]. Although Zhang et al. [13] did not discover a statistically significant difference in gonadotropin usage, the overall trend continues to support the resource-sparing potential of DYG-based PPOS regimens.

Hormonal Profiles

Both progestins demonstrated effective endocrine modulation. E2 and P levels followed expected trends in all groups, and both treatments achieved adequate pituitary suppression. Post-trigger LH and FSH levels varied slightly between the groups. Notably, MPA was associated with higher post-trigger LH levels in Zhang et al. [13] while Yu et al. [15] reported higher post-trigger FSH in the DYG group. Despite these biochemical differences, they did not translate into significant variations in clinical or embryological outcomes, suggesting that both agents build a hormonally favorable environment for embryo development and oocyte maturation.

Aligned with the findings of Lokshin et al. [9], both studies [13,15] confirmed that DYG and MPA effectively prevented premature LH surges, a key function of the PPOS protocol. Furthermore, they underscored that despite subtle endocrine variations, clinical efficacy remains comparable, supporting the interchangeable use of these agents based on patient or provider preference.

Pregnancy and Neonatal Outcomes

Pregnancy outcomes following FET cycles were also similar between groups. Clinical pregnancy rates, biochemical pregnancy rates, implantation, miscarriage, and ectopic pregnancy rates showed no significant differences across all three studies. This further supports the conclusion that both DYG and MPA are comparably effective in supporting pregnancy following PPOS.

While DYG showed a numerically lower miscarriage rate in Zhang et al. [13], the difference did not reach statistical significance. Yu et al. [15] and Huang et al. [14] also noted favorable but statistically non-significant trends in multiple pregnancy and miscarriage rates. These trends, while inconclusive, suggest that DYG may support slightly improved reproductive outcomes in some cases and warrant further exploration in larger RCTs.

Clinical Implications and Future Directions

Given its oral route, physiological hormone profile, and comparable efficacy, DYG represents a compelling alternative to MPA in PPOS protocols. Particularly for patients where reduced gonadotropin usage is a priority, such as those with cost concerns or poor tolerance to injectable medications, DYG may offer a more patient-friendly option. Additionally, the less intense pituitary suppression seen with DYG could be beneficial in patients requiring milder stimulation.

Nevertheless, the limited number of high-quality studies and variations in patient selection and stimulation protocols across studies highlight the urgency for further randomized controlled, well-designed trials. Future studies should explore subgroup analyses based on ovarian reserve and infertility etiology to tailor progestin selection more precisely to individual patient profiles.

## Conclusions

DYG emerged as a safe and effective alternative to MPA in PPOS protocols. Both agents demonstrated comparable efficacy in suppressing premature LH surges, supporting follicular development, and achieving similar pregnancy and neonatal outcomes. Additionally, DYG may offer advantages such as lower gonadotropin requirements and a more physiologic endocrine profile, making it a patient-friendly option, particularly in resource-conscious settings or among patients with heightened sensitivity to injectable medications.

While current evidence supports the clinical interchangeability of DYG and MPA, further high-quality RCTs are necessary to validate these findings. Future research should aim to clarify their respective roles in specific subpopulations, such as those with diminished ovarian reserve or distinct infertility etiologies, to optimize individualized treatment approaches in ART.
